# Clinical Application of CYP2C19 Pharmacogenetics Toward More Personalized Medicine

**DOI:** 10.3389/fgene.2012.00318

**Published:** 2013-02-01

**Authors:** Su-Jun Lee

**Affiliations:** ^1^Department of Pharmacology, Pharmacogenomics Research Center, Inje University College of Medicine, Inje UniversityBusan, South Korea

**Keywords:** CYP2C19, functional genetics, personalized medicine, SNP, drug

## Abstract

More than 30 years of genetic research on the *CYP2C19* gene alone has identified approximately 2,000 reference single nucleotide polymorphisms (rsSNPs) containing 28 registered alleles in the P450 Allele Nomenclature Committee and the number continues to increase. However, knowledge of *CYP2C19* SNPs remains limited with respect to biological functions. Functional information on the variant is essential for justifying its clinical use. Only common variants (minor allele frequency >5%) that represent CYP2C19*2, *3, *17, and others have been mostly studied. Discovery of new genetic variants is outstripping the generation of knowledge on the biological meanings of existing variants. Alternative strategies may be needed to fill this gap. The present study summarizes up-to-date knowledge on functional CYP2C19 variants discovered in phenotyped humans studied at the molecular level *in vitro*. Understanding the functional meanings of CYP2C19 variants is an essential step toward shifting the current medical paradigm to highly personalized therapeutic regimens.

## Introduction

Pharmacogenetics incorporates genetic information into clinical decision making to avoid adverse drug effects and improve drug efficacy. Drug responses can be affected by three major factors: pharmacokinetics, pharmacodynamics, and the underlying molecular mechanisms of disease. Because the DNA sequence of *CYP2C19* is highly polymorphic, this may account for much of the variability in the pharmacokinetics of drugs metabolized by CYP2C19. CYP2C19 metabolizes a number of drugs, including the antiulcer drug omeprazole (Andersson et al., 1992), the antiplatelet drug clopidogrel (Mills et al., 1992), the anticonvulsant mephenytoin (Andersson et al., 1992; Bertilsson, 1995), the antimalarial drug proguanil (Helsby et al., 1991; Ward et al., 1991), the anxiolytic drug diazepam (Bertilsson, 1995; Wan et al., 1996; Qin et al., 1999), and certain antidepressants such as citalopram (Sindrup et al., 1993), imipramine (Skjelbo et al., 1991), amitriptyline (Bouman et al., 2011), and clomipramine (Nielsen et al., 1994). The phenotype of CYP2C19 metabolic capacity can be categorized based on genotypes and includes extensive metabolizers (EM, two wild-type functional alleles), intermediate metabolizers (IM, two reduced functional alleles or one null allele and a functional allele), and poor metabolizers (PM, two non-functional alleles) of drugs. Undesirable side effects such as prolonged sedation and unconsciousness have been observed after administration of diazepam in CYP2C19 PMs (Bertilsson, 1995). In addition, a diminished response to the antiplatelet drug clopidogrel has been found in CYP2C19 PMs (Hulot et al., 2006; Brandt et al., 2007; Mega et al., 2009). However, proton pump inhibitor drugs, including omeprazole and lansoprazole, exhibit a greater cure rate for gastric ulcers with *Helicobacter pylori* infections in PMs than in EMs due to higher plasma concentrations of the parent drugs in PMs (Sohn et al., 1997; Furuta et al., 1998). In any case, clinical decision strategies following *CYP2C19* genotyping suggest two regimens: an adjustment of the drug dose according to the genotype or an alternative drug choice. However, the greatest uncertainty is in the IM group, in which interindividual variation is clearly observed. The underlying mechanism for this variation remains unclear. Integration of other factors, such as clinical factors, environmental factors, and drug response-modulating factors may be needed to understand this variation. A successful launch of personalized medicine in relation to CYP2C19 drugs would be impossible without resolving the variation in the IM group. The majority of *CYP2C19* pharmacogenetic studies have been conducted using CYP2C19*2, *3, and *17 variants. This article presents a collection of functional *CYP2C19* variants evidenced in human studies and discusses their utilities and limitations for clinical use.

## Diverse CYP2C19 Functional Variants

Four *CYP2C* genes have been identified in humans: *CYP2C8*, *CYP2C9*, *CYP2C18*, and *CYP2C19*. Among them, *CYP2C19* is the most polymorphic enzyme, and it metabolizes many important clinical drugs. Its activity can be inhibited by fluoxetine (Jeppesen et al., 1996), fluvoxamine (Jeppesen et al., 1996; Yao et al., 2003), lansoprazole, pantoprazole (Li et al., 2004), and ticlopidine (Tateishi et al., 1999), and can be induced by phenobarbital and rifampin (Madan et al., 2003). A number of *CYP2C19* polymorphisms have been identified. However, defining the quantitative value of altered functionality of the identified variant, such as the unequivocal percent value of wild-type activity, is difficult except for null alleles. One reason for this difficulty is from the different functional assay systems that include various molecular techniques with lab-to-lab variations. For example, to study protein-coding variants, enzyme expression systems have included yeast (Ferguson et al., 1998), baculovirus (Mankowski, 1999), and *Escherichia coli* (Lee et al., 2009), and have depended on the preference or experimental conditions of the laboratory. All systems are useful biochemical tools for determining relative activity compared to wild-type activity *in vitro*. However, results of comparative parameters are not identical among the assay systems. In addition, not all *in vitro* assay systems reflect the same phenomena of those conditions *in vivo*. Therefore, a quantitative comparison of *CYP2C19* variants with wild-type *CYP2C19* has been difficult and should be interpreted with great caution.

New *CYP2C19* variants have been discovered by direct DNA sequencing using two kinds of human DNA samples, normal healthy subjects or phenotyped individuals. *CYP2C19* variants identified by DNA sequencing in individuals that have been characterized by clinicians as PMs or outliers include CYP2C19*2, *3, *4, *5, *6, *7, *8, *16, *17, and *26, all based on comparisons to wild-type CYP2C19 activity *in vivo* or in *in vitro* functional studies, with the exception of CYP2C19*16 (Wilkinson et al., 1989; Balian et al., 1995). Briefly, CYP2C19*2 is the most common variant, which is a single base pair mutation in exon 5 (G > A), resulting an aberrant splice site (de Morais et al., 1994b). Creation of this splice site alters the reading frame of the mRNA beginning at amino acid 215 and produces a premature stop codon of 20 amino acids downstream (de Morais et al., 1994b); in that study, 7 of 10 Caucasians and 10 of 17 Japanese PMs for mephenytoin were homozygous for this mutation. CYP2C19*3 has been found in Japanese PMs that were not homozygous for CYP2C19*2 (de Morais et al., 1994a). CYP2C19*3, the result of the 636G > A mutation in exon 4, creates a premature stop codon. CYP2C19*2 and *3 are responsible for the majority of PM phenotypes in the metabolism of CYP2C19 substrate drugs (Goldstein, 2001; Xie et al., 2001). CYP2C19*4, an A > G mutation in the initiation codon, was identified in Caucasian PMs of mephenytoin (Ferguson et al., 1998); in that study, CYP2C19*4 cDNA was not expressed into the protein in a yeast expression system or an *in vitro* translation assay, whereas the CYP2C19*1 cDNA was expressed and translated in both systems, suggesting a new PM allele. CYP2C19*5, a 1297C > T mutation in the heme-binding region, results in a Arg433Trp substitution and has been identified in a single Chinese PM of *S*-mephenytoin (Xiao et al., 1997) and 1 in 37 white PMs of *S*-mephenytoin (Ibeanu et al., 1998a). A recombinant enzyme activity study indicated that this allele abolishes the activity toward *S*-mephenytoin and tolbutamide, suggesting a PM allele (Ibeanu et al., 1998a). CYP2C19*6, a 395G > A mutation in exon 3, leads to an Arg132Gln substitution and has been found in a white PM outlier of mephenytoin (Ibeanu et al., 1998b). In this study, recombinant protein of CYP2C19*6 prepared in an *E. coli* expression system exhibited negligible activity toward *S*-mephenytoin compared to that of wild-type, indicating that the CYP2C19*6 allele contributes to the PM phenotype in whites. CYP2C19*7 is a T > A mutation at the 5′ donor splice site of intron 5 and results in a PM allele. This allele has been found in a mephenytoin PM outlier of a Danish individual (Ibeanu et al., 1999). CYP2C19*8, a T358C change in exon 3 that results in a Trp120Arg substitution, was first identified in a French subject enrolled in a cancer risk study with mephenytoin activity (Benhamou et al., 1997). In a recombinant study, the CYP2C19*8 protein exhibited a 90% decrease in activity for *S*-mephenytoin and a 70% reduction in tolbutamide activity compared to wild-type (Ibeanu et al., 1999). CYP2C19*16 is a 1324C > T change in exon 9 located close to the heme-binding region that results in a Arg442Cys substitution, and was first identified in a Japanese individual with a low capacity to metabolize mephobarbital (Morita et al., 2004). Because this amino acid change is located close to the heme-binding site, it is proposed to have decreased activity for CYP2C19 substrate drugs. CYP2C19*17 is an allele carrying −806C > T and −3042C > T found in the 5′ regulatory region (Sim et al., 2006). Individuals with CYP2C19*17/*17 exhibit 35–40% lower omeprazole area under the plasma concentration-time curve values than the individuals having CYP2C19*1/*1, suggesting that this allele leads to an increased metabolizer phenotype. In Sim et al. (2006), a reporter assay showed increased transcriptional activity of CYP2C19*17 and electrophoretic mobility shift assays showed specific binding of human hepatic nuclear proteins to an element carrying −806T but not −806C. CYP2C19*26, a 766G > A change in exon 5 resulting in a D256N substitution, was first identified in an omeprazole PM outlier of a Vietnamese individual; a recombinant enzyme activity assay prepared in an *E. coli* expression system showed a significant decrease in *V*_max_ for omeprazole (2.6-fold) and mephenytoin metabolism (twofold; Lee et al., 2009).

**Figure 1:**
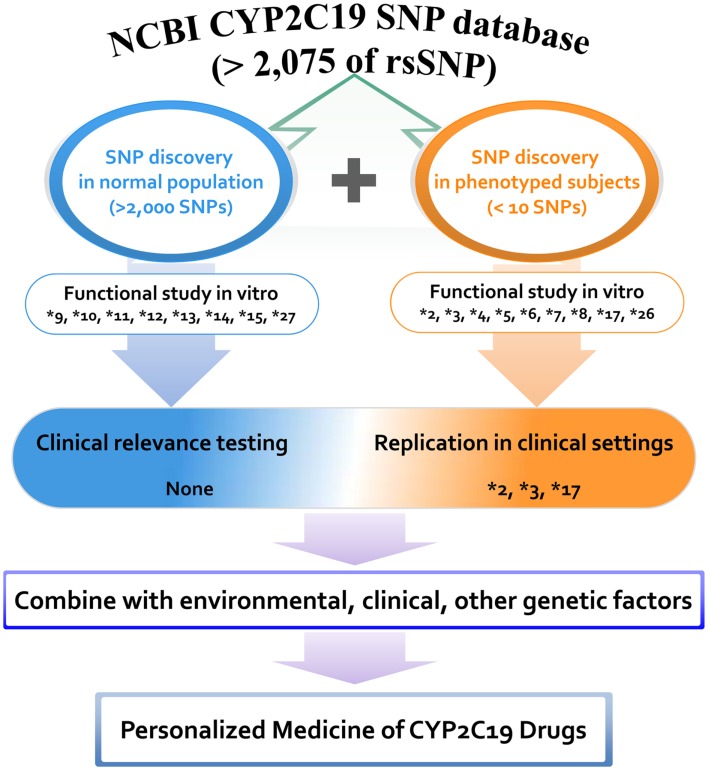
**Distribution of *CYP2C19* variants discovered and characterized in phenotyped human subjects and normal populations**. A total of 2,075 rsSNPs have been reported in NCBI CYP2C19 SNP database at the present stage. Among them the number of SNPs that are functionally studied *in vitro* and validated *in vivo* is limited. Proposed scheme is for the personalized medicine of CYP2C19 substrate drugs.

The majority of other alleles have been discovered in various human projects by using DNAs obtained from normal populations. *CYP2C19* variants that were discovered without phenotyping but which were characterized by *in vitro* systems include *9, *10, *11, *12, *13, *14, *15, and *27. A positive correlation between *in vitro* data and *in vivo* biological evidence would strengthen the incorporation of genetic data into clinical practice. Although more than 2,000 variants have been identified in the last several decades, alleles characterized via either *in vivo* or *in vitro* studies are limited to less than 20 variants, indicating a huge gap between the number of discovered single nucleotide polymorphisms (SNPs) and the number of functionally known SNPs. A summary of *CYP2C19* variants that have been discovered and characterized in phenotyped human subjects is presented in Figure 1. Clinical application of variant alleles with functional information is critical in the clinical setting for individualized drug therapy. Profiling of rare variants reflecting interindividual variability in drug responses could be difficult, but is needed for incorporating *CYP2C19* pharmacogenetics into the clinical decision making process.

Because CYP2C19*2 and CYP2C19*3 are the most common and defective variants (Goldstein et al., 1997; Xie et al., 2001), a number of reports have described the clinical significance of these variants. The promoter variant CYP2C19*17 has been shown to be associated with increased CYP2C19 activity due to increased transcription of *CYP2C19*. Therefore, homozygous carriers of CYP2C19*17 exhibit rapid clearance of CYP2C19 substrate drugs compared to wild-type (Sim et al., 2006; Ingelman-Sundberg et al., 2007; Rudberg et al., 2008). As a consequence, individuals with CYP2C19*17 are likely to exhibit a lack of response to certain PPIs and antidepressants compared to EMs (CYP2C19*1/*1); this is due to rapid clearance of the drugs. Although most variation in the drug response can be explained by these three variants (CYP2C19*2, *3, and *17), high interindividual variation in subjects homozygous for CYP2C19*1 has also been observed (Sim et al., 2006). It is well-known that CYP2C19 activity is influenced by clinical factors, various inducers and inhibitors, drug–drug interactions, and other genetic polymorphisms in drug response-related genes. Because most studies have translated drug response data after genotyping for common alleles only, variants that have not been detected may be missing. More candidate SNPs may improve the translational process for drug responses. The limited amount of genotyping in current research may be due to the lack of data on the functional significance (*in vitro* or *in vivo*) of the allele and the low or undetermined allele frequency in the study population. Therefore, information on the functional significance of the variant allele would help researchers to include more candidate SNPs for genotyping in their clinical studies. This would ultimately improve translational quality in clinical settings and would be a beginning step for launching personalized medicine because physicians could easily communicate with patients using biological evidence for the drug response prediction instead of explaining the statistical risk without biological significance.

## Current Clinical Utilities of *CYP2C19* Polymorphisms

Many clinical drugs are metabolized by CYP2C19, and hence, the pharmacokinetics of CYP2C19 substrate drugs is influenced by *CYP2C19* polymorphisms. Altered CYP2C19 activity can be, at least in part, predicted by the *CYP2C19* genotype. The most extensively used polymorphic variants for genotype and phenotype association studies have been CYP2C19*2, *3, and *17 (Goldstein, 2001; Xie et al., 2001; Desta et al., 2002). Ethnic differences in the frequency of CYP2C19*2, *3, and *17 alleles are summarized in Table 1. Identification of PM alleles or the CYP2C19*17 variant in patients and the modification of therapy regimens does not represent personalized drug therapy but is useful for the prevention of side effects. Because the human genome is very difficult to understand and drug responses are affected by several modulating genes with environmental factors, the development of personalized medicine for CYP2C19 substrate drugs using only *CYP2C19* and other modulating gene’s genotypes is nearly impossible. However, several FDA-approved drugs are strongly affected by *CYP2C19* genotypes (see Table 2), and their labels include pharmacogenomic information (http://www.fda.gov/Drugs/ScienceResearch/ResearchAreas/Pharmacogenetics). Briefly, diazepam is demethylated by CYP2C19 (Jung et al., 1997), and hence its pharmacokinetics are affected by *CYP2C19* genetic polymorphisms. The plasma half-life of diazepam is approximately fourfold longer in individuals with PM genotypes than in individuals homozygous for wild-type CYP2C19*1 (Wan et al., 1996; Qin et al., 1999). PMs may be at risk for toxic doses of diazepam and therefore more care is required to determine the diazepam dose for such subjects, especially for certain ethnicities because the frequency of PM differs between ethnic groups, being 3–6% in whites and blacks, 13–23% in Asians, and 38–79% in Polynesians and Micronesians (Kaneko et al., 1999; Xie et al., 2001). The *CYP2C19* genotype affects the metabolism of PPIs, resulting in altered cure rates for *H*. *pylori* infection in peptic ulcer patients. In a previous study, the cure rate of peptic or duodenal ulcers for Japanese patients that received dual therapy with omeprazole (20 mg/day for 2 weeks) and amoxicillin (2,000 mg/day for 2 weeks) was 100% in *CYP2C19* PMs, 60% for those who were heterozygous for one mutant allele, and 29% in individuals homozygous for the CYP2C19*1 allele (Furuta et al., 1998). These differences are attributable to the *CYP2C19* PM genotype’s impaired metabolism of PPIs, which leads to higher PPI plasma concentrations in PM individuals (Furuta et al., 2004). The contribution of *CYP2C19* to the metabolism of various PPIs differs. For example, in terms of plasma drug concentration-time curves, the ratio of the area under the curve of PMs versus EMs decreases in the following order: omeprazole, pantoprazole, lansoprazole, and rabeprazole (Funck-Brentano et al., 1997; Furuta et al., 2004). This is in agreement with the fact that the cure rate of rabeprazole is less dependent on the *CYP2C19* genotype compared to other PPIs. Patients with CYP2C19*17 exhibit an enhanced response to clopidogrel with an increased risk of bleeding due to

**Table 1:** Frequencies of CYP2C19 *2, *3, and *17 alleles in different ethnic populations.

Ethnic group	Allele no.	*2 (681G > A)	*3 (636G > A)	*17 (−806C > T)	Reference
		Splicing defect	W212X	Increased transcription	
**WHITE**					
Faroese	622	0.187	N.D	0.154	Pedersen et al. (2010)
Danish	552	0.150	N.D	0.201	Pedersen et al. (2010)
French	48	0.208	N.D	0.188	Berge et al. (2011)
Italian	720	0.094	0.008	N.D	Scordo et al. (2001)
Polish	250	0.116	N.D	0.272	Kurzawski et al. (2006)
Norwegian	664	0.181	0.006	0.220	Rudberg et al. (2008)
Sweden	370	0.160	N.D	0.200	Ramsjö et al. (2010)
Russians	580	0.114	0.003	N.D	Gaikovitch et al. (2003)
Caucasian	284	0.136	0.000	0.201	Myrand et al. (2008)
**BLACK**					
African-American	216	0.250	0.000	N.D	Bravo-Villalta et al. (2005)
African-American	472	0.182	0.008	N.D	Luo et al. (2006)
African-American	228	N.D	N.D	0.210	Kearns et al. (2010)
Ethiopian	380	N.D	N.D	0.179	Sim et al. (2006)
Nigerian	86	0.151	0.000	N.D	Babalola et al. (2010)
Egyptians	494	0.110	0.002	N.D	Hamdy et al. (2002)
**ASIAN**					
Korean	542	0.284	0.101	0.015	Kim et al. (2010)
Korean	100	0.290	0.050	0.020	Lee et al. (2009)
Chinese	136	N.D	N.D	0.044	Sim et al. (2006)
Chinese	800	0.247	0.033	0.012	Chen et al. (2008)
Japanese	530	0.279	0.128	0.013	Sugimoto et al. (2008)
Japanese	200	0.345	0.090	0.005	Myrand et al. (2008)
India	40	0.375	0.000	N.D.	Lamba et al. (2000)
India	906	0.350	0.010	N.D.	Jose et al. (2005)
Vietnamese	330	0.264	0.049	N.D.	Lee et al. (2007)
Thai	1548	0.290	0.030	N.D.	Tassaneeyakul et al. (2006)
Burmese	254	0.300	0.040	N.D.	Tassaneeyakul et al. (2006)
Karen	262	0.280	0.010	N.D.	Tassaneeyakul et al. (2006)
Jordanian	156	0.160	0.000	N.D.	Zalloum et al. (2012)
Iranians	206	0.120	0.100	N.D.	Hashemi-Soteh et al. (2012)

*N.D., not determined*.

**Table 2:** **Genetic polymorphisms in *CYP2C19* and their clinical consequences**.

Drug	Therapeutic area	Clinical consequences
Diazepam	Psychiatry	Increased risk of sedation time and unconsciousness in PM genotype due to the prolonged half-life of diazepam
Omeprazole, lansoprazole	Gastroenterology	Increased cure rates due to increased half-life of the parent drugs in PM genotypes
		Decreased cure rates in the EM genotype
Clopidogrel	Cardiovascular	Decreased response to clopidogrel in the PM genotype due to low transformation into active metabolite and increased risk of recurrent MI, stroke, and stent thrombosis
		Increased risk of bleeding disorder in individuals homozygous for the CYP2C19*17 allele due to increased inhibition of platelet function

*PM, poor metabolizer; EM, extensive metabolizer; MI, myocardial infarction*.

the higher rate of biotransformation into the active metabolite (Sibbing et al., 2010). In addition, an improved protective effect of clopidogrel after myocardial infarction has been observed in patients carrying the CYP2C19*17 allele (Tiroch et al., 2010). The major enzymes involved in the production of the active metabolite of clopidogrel have been identified as CYP2C19, CYP3A4, and paraoxonase-1 (Clarke and Waskell, 2003; Bouman et al., 2011), although clopidogrel itself is a potent inhibitor of CYP2C19 and CYP3A4 (Richter et al., 2004). In general, patients that carry one or two CYP2C19 loss-of-function alleles exhibit diminished platelet inhibition after clopidogrel treatment compared to those with the EM genotype (Hulot et al., 2006; Brandt et al., 2007; Mega et al., 2009; Kubica et al., 2011). CYP2C19 PMs may not benefit from clopidogrel and thus alternative drugs, such as prasugrel, should be considered. No definitive recommendations have been established regarding dose adjustment of clopidogrel based on *CYP2C19* genotype testing. This may be due to the fact that other downstream genes, such as GPIIb/IIIa receptor genes, and other modulating factors can render a patient more sensitive or more resistant to clopidogrel despite having the same genotype. Larger-scale investigations using genotype-guided clopidogrel therapy compared to other therapy options would reveal the effectiveness of *CYP2C19* genotype testing for clopidogrel therapy.

## Future Directions for Clinical Application

To date, the pharmacogenetic data on CYP2C19 clearly support that genetic variants alter the drug responses of its substrate drug. However, clinical application of CYP2C19 pharmacogenetics is limited to certain genotypes, mostly null alleles, and functionally known variants with a minor allele frequency of >5%. Clinical research using common variants only in case and control groups can enhance the statistical power of the evidence for that variant. However, the number of study populations that would benefit from pharmacogenomics research would be greatly reduced if such studies focused on common variants for strong statistical evidence. One goal of pharmacogenomics is to provide personalized medicine to each individual patient and to provide him or her an appropriate dose of the most appropriate drug. Therefore, more diversified investigations or strategies including low-frequency variants are needed to develop a standard practice for human applications. This may be ineffective or unrealistic for personalized medicine if clinical trials wait for the enrollment of a certain number of participants carrying the target genotype to satisfy the statistical power when assessing the role of that particular variant. The biological function of most rare variants remains unknown or has not been investigated *in vitro*, which has made physicians reluctant to test these individuals in clinical trials. Therefore, more data on functional genomics are needed particularly for rare variants. Although CYP2C19 is a major enzyme for the metabolism of certain drugs and influences their pharmacokinetics, there are many other genes that can modulate or mask the genetic effect of CYP2C19 polymorphisms, including genes involved in pharmacodynamics. Fortunately, recent whole genome sequencing and exome sequencing data have identified numerous variants, and these findings have led to genome-wide association studies using dense genomic markers on chips, resulting in the discovery of new determinants of drug responses. In conclusion, the translation of CYP2C19 pharmacogenetics into clinical practice is currently limited to a small number of functional variants, although more than 2,000 variants have already been discovered. More comprehensive and diverse research covering a large number of CYP2C19 variants will lay the foundation for improved personalized medicine in the future.

## Conflict of Interest Statement

The author declares that the research was conducted in the absence of any commercial or financial relationships that could be construed as a potential conflict of interest.
